# An Unmitigated Quack

**Published:** 1875-09

**Authors:** 


### An Unmitigated Quack.

Letters have been received by members of the Faculty of the Medical Department of the University of Buffalo in regard to the following, taken from a Leavenworth, Ka., paper.

DR. C. P. MARSHALL, Physician-in-Chief of Throat and Lung Department of the Buffalo City Hospital, and Professor of the Diseases of the Throat and Lungs, in the Medical Department of the Buffalo, N. Y. University, is now in our city, on his return from Colorado, and the Rocky Mountain district, which he has been visiting, with a number of his patients, to test the curative power of that congenial clime in consumptive cases.

The doctor is stopping with his patients for a few days at the Mansion House, where, he states, he would be happy to see, converse with, and examine, *free of charge*, any of the citizens of this community, who may be suffering with any throat or lung complaint, and will most cheerfully give them the benefit or his experience with the climate of Colorado in the cure of all their maladies, besides many useful hints that may prove of great benefit. All are cordially invited to give him a call.—*Leavenworth Daily Times, September* 3, 1875.

The Dr. (?) Marshall referred to came to Buffalo some time since claiming to have been Surgeon-in-Chief to the Brooklyn City Hospital and to have been connected with various Medical Institutions in New York and Brooklyn. His boastful claims and extensive advertising were sufficient evidence of their lack of truth. He was, however, vouched for, we believe, by an Editor of the *Sunday News* and doubtless caught a few dupes. Of his success as a practitioner we have no knowledge, we only know that some of his creditors would be most happy to see him. We believe he has not yet returned from that Rocky Mountain tour and infer that he will not do so for some time to come. It remains to be seen whether the paper which devoted so many columns to his laudations will be willing to occupy a few lines with an account of his efforts to gain notoriety and cash upon false pretenses.

